# Metabolic reprogramming in clear cell renal cell carcinoma: core pathways and targeted therapeutic strategies

**DOI:** 10.3389/fgene.2025.1752384

**Published:** 2026-01-05

**Authors:** MingWei Zhan, BinBin Zhao, Haote Chen, Junjie Wu, Run Shi, Feng Gao, Lin Zhao, Jingyu Zhu

**Affiliations:** 1 Department of Urology, Hangzhou TCM Hospital of Zhejiang Chinese Medical University (Hangzhou Hospital of Traditional Chinese Medicine), Hangzhou, China; 2 Department of Urology, Hangzhou Integrative Medicine Hospital Affiliated to Zhejiang Chinese Medical University (Hangzhou Red Cross Hospital), Hangzhou, China; 3 Department of Urology, Jinling Clinical Medical College, Nanjing University of Chinese Medicine, Nanjing, China; 4 Department of Oncology, The First Affiliated Hospital of Nanjing Medical University, Nanjing, China

**Keywords:** belzutifan, clear cell renal cell carcinoma (ccRCC), DCCD, ferroptosis, glutamine metabolism, immunometabolism, lipid droplets/PLIN2, one-carbon metabolism

## Abstract

Clear cell renal cell carcinoma (ccRCC), rooted in VHL loss and dysregulated HIF signaling, is defined by a sweeping metabolic overhaul: intensified glycolysis, a “downshifted” TCA cycle, the buildup of lipid droplets and cholesteryl esters, and a pronounced dependence on glutamine and one-carbon metabolism—all tightly intertwined with an immunosuppressive microenvironment. Drawing on single-cell and spatial multi-omics, metabolomic and lipidomic profiling, and imaging-based evidence, this article maps the critical nodes of carbon, lipid, amino-acid, and one-carbon pathways, and their crosstalk with ferroptosis. It highlights how metabolic heterogeneity—exemplified by the DCCD spectrum—shapes prognosis and therapeutic response. The review further synthesizes how metabolic–immune coupling, including lipid metabolic rewiring in TAMs and MDSCs, and lactate/lipid stress in CD8^+^ T cells, contributes to immune-therapy resistance. On the translational front, HIF-2α inhibitors (such as belzutifan), strategies that suppress or oxidize lipids to trigger ferroptosis, and interventions targeting glutamine and one-carbon metabolism show promise when rationally combined with ICIs, TKIs, or anti-angiogenic therapies. We propose a stratified decision framework anchored in DCCD state, lipid-droplet/PLIN2 phenotype, ferroptosis sensitivity, and HIF activity, and discuss the emerging roles of radiopathomics (e.g., CT HU–PLIN2 coupling) and circulating metabolic fingerprints in companion diagnostics. Looking toward clinical deployment, advancing standardization within MSI/IBSI and FAIR data principles—and launching biomarker-enriched, prospective multicenter trials—will be essential to demonstrate the real-world value of precision metabolic oncology in the personalized treatment of ccRCC.

## 1 Introduction

Clear cell renal cell carcinoma (ccRCC) is the most common histological subtype of renal cell carcinoma (RCC), accounting for approximately 80% of RCC cases. Kidney cancer represents about 2–2.4% of all cancers worldwide (Wild et al., 2020; Nezam et al., 2024). According to the latest GLOBOCAN estimates, kidney cancer results in over 430,000 new cases and approximately 180,000 deaths each year, highlighting its substantial global disease burden (Sung et al., 2021). It is more common in men aged 60–70. The vast majority (>95%) are sporadic solitary tumors, and a few (about 5%) are related to genetic diseases such as VHL syndrome (Nezam et al., 2024). The main risk factors include obesity, hypertension, smoking, and long-term dialysis (especially those with acquired cystic nephropathy), among which obesity and hypertension suggest the potential effects of lipid metabolism disorders and energy homeostasis imbalance (Wild et al., 2020). The diagnosis of ccRCC usually relies on imaging and histopathological evaluation. Still, because of the lack of early symptoms, many patients are already in the late stages of diagnosis, resulting in a poor prognosis (Wild et al., 2020). Therefore, an in-depth understanding of its molecular mechanism and metabolic characteristics is of great significance for early diagnosis and targeted treatment.

The occurrence of ccRCC is closely associated with a VHL (Von Hippel-Lindau) gene mutation. The inactivation of the VHL gene leads to the stabilisation of HIF-2α, activates the expression of downstream genes, and promotes the proliferation and survival of tumor cells (Zh et al., 2023). In histology, ccRCC is characterised by cytoplasmically transparent tumor cells, mainly due to the accumulation of large amounts of glycogen, phospholipids, and neutral lipids, especially cholesterol esters (Wang et al., 2025). The accumulation of these lipids is not only a pathological feature of ccRCC but also closely associated with its metabolic reprogramming. Metabolic reprogramming is one of the key mechanisms by which tumor cells adapt to rapid proliferation and a harsh microenvironment. In ccRCC, loss of function of the VHL/HIF pathway leads tumor cells to sustain elevated glycolysis under hypoxic conditions, similar to the Warburg effect (Zh et al., 2023). In addition, lipid metabolism has also changed significantly. Tumor cells promote the accumulation of lipid droplets by ingesting exogenous lipids and enhancing endogenous lipid synthesis, and provide support for cell membrane synthesis and energy storage (Fresnedo et al., 2025). These metabolic changes support tumor cell growth and survival and promote immune escape by altering the function of immune cells in the tumor microenvironment (TME) (Deng et al., 2025).

In recent years, multi-group research has revealed the complexity of ccRCC metabolic reprogramming. Single-cell and spatial transcriptomic analyses reveal extensive metabolic heterogeneity in ccRCC tumors; the metabolic features of distinct regions are likely indicative of tumor aggressiveness and treatment susceptibility (Hu et al., 2024). These results provide a novel view of ccRCC metabolic features and lay the foundation for tailoring an effective therapeutic regimen.

## 2 Core pathways and molecular basis of metabolic reprogramming

### 2.1 VHL–HIF axis-driven glucose/carbon metabolism pathway reprogramming

VHL gene mutation or inactivation is one of the most common molecular characteristics of ccRCC. The loss of VHL function leads to the stabilisation of HIF-1α and HIF-2α (Cancer Genome Atlas Research Network, 2013; Jaakkola et al., 2001; Bao et al., 2019). HIF is an oxygen-sensitive transcription factor that helps tumor cells adapt to a hypoxic environment by upregulating a series of genes. Specifically, HIF-1/2α promotes glucose uptake in tumor cells, acidification of the tumor environment, and the formation of new blood vessels by upregulating genes such as GLUT1, CA9, and VEGF, and supports tumor growth (Zh et al., 2023; Almanzar et al., 2025; Reinfeld et al., 2022). In addition, HIF-1/2α interacts closely with mTOR, MYC, PI3K/AKT, and other signalling pathways, jointly regulating multiple levels of tumor metabolism, including glycolysis, fatty acid synthesis, and amino acid metabolism (Badoiu et al., 2023; Yecies and Manning, 2011; Li et al., 2020).

For the first-in-class HIF-2α inhibitor belzutifan, the process has been very rapid, first a flurry of studies, and now clinical trials. The latest clinical data show that belzutifan can effectively improve progression-free survival (PFS) and symptom relief in patients with ccRCC by inhibiting HIF-2α activity, especially when combined with other targeted drugs (Jonasch et al., 2021; Choueiri et al., 2024; Song et al., 2024).

### 2.2 Carbon metabolic pathway: glycolysis enhancement and mitochondrial function inhibition

A key aspect of metabolic reprogramming is the Warburg effect: in the presence of oxygen, tumor cells preferentially metabolise glucose through glycolysis instead of oxidative phosphorylation to generate energy. ccRCC cells enhance the glycolysis pathway to facilitate glucose uptake, lower the pH in tumor cells by lactic acid accumulation, and support a beneficial environment for their growth (Zh et al., 2023; Yang et al., 2023).

In ccRCC, tumor cells suppress the TCA cycle and modify mitochondria to adapt to heightened metabolic demands. This metabolic transformation is intimately associated with immunosuppression, drug resistance, and metastasis. Research suggests that the Warburg effect not only supports tumor cell growth but also suppresses immune cell function through changes in the TME metabolic milieu (e.g., lactic acid and hypoxia), thereby increasing resistance to immune checkpoint inhibitors (ICI) and chemotherapeutic agents (Zh et al., 2023; Yang et al., 2025).

### 2.3 Metabolism-related pathways for lipid metabolism and lipid droplet formation

Lipid metabolism is a key component of reprogramming ccRCC metabolism. Cancer cells synthesize, store, and mobilize lipids to favour the formation of cell membranes and energy reserves in tumor cells. Lipid droplet and cholesterol ester accumulation are hallmarks of ccRCC, and tumor cells express critical enzymes driving lipogenesis, including fatty acid synthase (FASN), unsaturated fatty acid synthase (SCD), and long-chain lipoacyl-CoA synthase (ACSL) (Deng et al. 2025; Klasson et al., 2022; Li et al., 2023).

Lipid metabolism is closely linked to iron-dependent cell death (ferroptosis). The buildup of lipid peroxides triggers ferroptotic cell death, while unsaturated fatty acids and the GPX4/xCT pathways regulate this lipid-peroxide–driven process. Tumor cells cope with metabolic stress and determine their tolerance to treatment by regulating the activity of these pathways (Zh et al., 2023; Zhou et al., 2024).

### 2.4 Amino acid and one-carbon metabolism-associated pathways

Metabolism of amino acids is vital for the regulation of redox homeostasis, the supply of NADPH, and the synthesis of nucleotides in tumor cells. ccRCC cells show a strong dependency on glutamine and serine/one-carbon metabolism. Through these pathways, tumor cells can maintain their antioxidant ability and meet the needs of rapid proliferation (Zhang et al., 2025a; Kaushik et al., 2022; Lyu et al., 2025). In recent years, metabolic inhibition and synthetic lethal strategies have emerged as a new direction in tumor treatment. For example, drugs targeting glutamine metabolism, amino acid transport, and one-carbon metabolism have shown promising efficacy in early clinical trials, especially when combined with other therapies (Zh et al., 2023; Jin et al., 2023; Ren et al., 2024).

### 2.5 Metabolic heterogeneity and clonal evolution: the metabolic basis of tumor evolution

Single-cell and spatialomics techniques revealed high metabolic heterogeneity in ccRCC, reflecting differences across tumor regions and cell subgroups (Shi et al., 2025). In particular, there is a line called “de-lipid-droplet cell differentiation (DCCD)” in the tumor. These tumor cells have fewer lipid droplets but exhibit strong nutrient uptake, rapid proliferation, and a poor prognosis (Hu et al., 2024; Yang et al., 2024). The heterogeneity of metabolic states offers new opportunities for individualised treatment strategies and stratified approaches to overcome tumor drug resistance and immune escape. Table 1 further illustrates the key characteristics of each metabolic pathway and its therapeutic significance.

**TABLE 1:** Metabolic reprogramming in ccRCC: core pathways.

Core pathways	Key features	Implications on tumor metabolism	Therapeutic insights	References
The VHL–HIF axis integrates multiple signaling pathways involved in metabolic reprogramming	VHL gene mutation or deletion stabilises HIF-1α/2α; HIF upregulates GLUT1, CA9, and VEGF to promote glucose uptake, the formation of an acidic microenvironment, and tumor angiogenesis	HIF activation promotes tumor growth, immune escape, and treatment tolerance (e.g., ICI, chemotherapy)	The HIF-2α inhibitor belzutifan has a good prospect in ccRCC. Combined with other therapies, it can improve progression-free survival (PFS) and symptom relief	(Zh et al., 2023; Almanzar et al., 2025; Reinfeld et al., 2022)
Carbon metabolism: glycolysis and mitochondrial function	Warburg effect: glycolysis enhancement, lactic acid accumulation; ccRCC cell TCA cycle downregulation	Metabolic reprogramming leads to a decrease in immune cell activity and an increase in tolerance to immunotherapy	Targeted glycolysis and mitochondrial function are expected to enhance immune response and reduce treatment tolerance	(Zh et al., 2023; Yang et al., 2023; Yang et al., 2025)
Lipid metabolism: synthesis, storage, and mobilisation	Significant accumulation of lipid droplets; enhanced lipid metabolism (FASN, SCD, ACSL upregulation); associated with ferroptosis	Abnormal lipid metabolism promotes membrane construction and energy storage, affecting the survival and treatment response of tumor cells	Targeted lipid metabolism (e.g., FASN and SCD) can relieve immunosuppression and promote ferroptosis	(Deng et al. 2025; Klasson et al., 2022; Li et al., 2023; Zhou et al., 2024)
Amino acids and one-carbon metabolism	Glutamine metabolism and serine/one-carbon metabolism maintain antioxidant capacity, which is crucial for tumor proliferation	Metabolic dependence provides opportunities for targeted treatment, including inhibition of glutamine and one-carbon metabolism	Targeted amino acid metabolism (especially the glutamine and serine pathways) shows early clinical potential for overcoming treatment tolerance	(Zhang et al., 2025a; Kaushik et al., 2022; Lyu et al., 2025; Jin et al., 2023; Ren et al., 2024)
Metabolic reprogramming and clonal evolution/heterogeneity	ccRCC shows significant metabolic heterogeneity: for example, DCCD spectrum lipid droplet content is low, nutrient uptake is strong, and prognosis is poor	The diversity of tumor metabolism within the body affects treatment stratification, underscoring the importance of individualised strategies	Metabolic stratification based on DCCD phenotype and lipid droplet levels is expected to guide individualised treatment plans	(Hu et al., 2024; Yang et al., 2024)

## 3 Manifestations of metabolic pathway features in the tumor microenvironment and medical imaging

### 3.1 Metabolism-immune interaction pathway and tumor immune microenvironment

The lipid-rich, hypoxic metabolic ecology of ccRCC will systematically “transform” the metabolic state and function of immune cells in the TME, which is a key driver of immunotherapy drug resistance (Niu et al., 2025a). The metabolic reprogramming of myeloid immune cells is one of the driving forces shaping the immunosuppressive microenvironment. Tumor-associated macrophages (TAM) tend to absorb a large amount of oxidative lipids released by tumor cells in ccRCC through scavenger receptors (such as CD36). These lipids drive mitochondrial β-oxidation in intracellular cells, promote their polarisation to an immunosuppressive M2 phenotype, and secrete IL-10, TGF-β, and other inhibitory cytokines (Hu et al., 2024; Simeth et al., 2025). In other urinary system tumors, transcriptomic studies centred on macrophages have revealed an immune-regulatory mechanism driven by lipid metabolism, which also supports the existence of similar metabolic-immune mechanisms in ccRCC (Wang et al., 2023; Wang et al., 2024). Myeloid-derived suppressor cells (MDSCs) use fatty acid metabolism to maintain their survival and inhibitory functions, and directly inhibit T cell proliferation and activity by consuming essential amino acids, such as arginine and cysteine, in the microenvironment (Zhang et al., 2025b).

In the T cell compartment, the function of CD8^+^ T cells is suppressed by multiple mechanisms, including lactic acid accumulation: the high lactic acid environment produced by tumor glycolysis directly inhibits mTOR signalling and T cell function (Brand et al., 2016; Watson et al., 2021). Lipid coercion: Excessive lipids in the TME lead to mitochondrial dysfunction in T cells and upregulate markers of T cell depletion, such as PD-1 (Fadini, 2020; Lin et al., 2020). Nutritional competition: The competition between tumor cells for glucose and glutamine causes T cells to “lack metabolism” (Cha et al., 2015; Ho et al., 2015).

These metabolic mechanisms together lead to T cell depletion and apoptosis, significantly weakening the efficacy of immune checkpoint inhibitors (ICI). It is worth noting that tumor lipid metabolism is systematically negatively correlated with the infiltration density and functional status of CD8^+^ T cells (Simeth et al., 2025; Zhang et al., 2025b). Therefore, targeting abnormal lipid metabolism in tumor and medullary cells, or relieving T-cell metabolic stress through metabolic regulators (such as LDHA inhibitors and PPARα agonists), has become a promising strategy to improve the efficacy of ICI.

### 3.2 Integrated analysis of metabolomics and radiopathology-metabolism (Radiopathomics)

Non-invasive “metabolic fingerprints” provide a new tool for the clinical management of ccRCC. In body fluid metabolomics, plasma- or urine-based metabolite analysis has shown great potential. Using mass spectrometry and machine learning, markers including succinic acid, acylcarnitine, and specific phospholipids have been identified. These molecular characteristics can distinguish ccRCC from healthy individuals with high accuracy, and a multi-centre cohort has validated their reliability for early diagnosis and risk stratification (Ho et al., 2015; Huang et al., 2025; Ossolińska et al., 2025). At the level of image integration, the radiopathomics framework deeply correlates CT image information with molecular metabolic phenotype. The core finding is a significant negative correlation between the tumor lipid attenuation (HU value) in CT imaging and the expression level of the lipid droplet marker PLIN2 in the tissue (Deng et al., 2025; Wang, 2023). This relationship enables clinicians to infer a tumor’s lipid metabolic status noninvasively using routine CT imaging.

At present, artificial intelligence is further promoting the transformation of this field. Using a training model to decode complex features (texture, shape, etc.) in CT images, key metabolic phenotypes, such as PLIN2 expression and ferroptosis sensitivity, can be predicted, and patients with “high-risk” metabolic characteristics (such as DCCD tendency) can be identified before treatment (Deng et al., 2025). In the end, the combination of “body fluid metabolism spectrum + image lipid reading” is being built into a powerful, non-invasive diagnostic support system.

## 4 Treatment opportunities: from pathway to scheme

### 4.1 Treatment strategies for directly targeting metabolic pathways

Hypoxic axis: With the emergence of the HIF-2α inhibitor belzutifan, the hypoxia-driven metabolic–angiogenic program can be pharmacologically inhibited for the first time. The phase III LITESPARK-005 trial demonstrated that in patients with advanced ccRCC previously treated with ICIs and anti-angiogenic therapy, belzutifan improved PFS and ORR compared with everolimus, without new safety signals (Choueiri et al., 2024; Powles et al., 2025). Lipid metabolic reprogramming is tightly linked to ferroptosis. The system x_c⁻ (xCT/SLC7A11)–GSH–GPX4 axis represents a key brake on ferroptosis. Emerging evidence supports strategies that suppress lipid storage and/or promote lipid peroxidation to induce ferroptosis, potentially in combination with immunotherapy and anti-angiogenic therapy to broaden the therapeutic window (Deng et al., 2025; He et al., 2024).

Amino acids and one-carbon metabolism: ccRCC’s dependence on glutamine and one-carbon metabolism provides an entry point for synthetic death; at the mechanism and early clinical level, targets such as GLS/transporters show the potential to reverse drug resistance and improve immune response, and be alert to compensatory pathways and systemic toxicity (Wang, 2023).

### 4.2 Combined strategies of immune, antivascular, and radiotherapy based on metabolic pathways

Metabolic remodeling, vascular “normalization,” and T-cell infiltration can form a synergistic cascade: inhibiting HIF–VEGF signaling and dysregulated lipid metabolism may reduce myeloid-mediated immunosuppression, improve perfusion and oxygenation, and enhance CD8+ T-cell effector function, thereby providing a metabolic rationale for synergy between TKIs/VEGF inhibitors and ICIs. On the other hand, the strategy of lipid suppression or induction of lipid peroxidation + ICI is expected to overcome immune tolerance by improving antigen presentation and immunoinflammatory microenvironment (Deng et al., 2025; Zhang et al., 2025b).

### 4.3 Biomarker development and patient stratification based on metabolic pathways

Multi-omics stratification is being formed: integrated analysis of transcriptome–lipidome–immune infiltration reveals that there is a coupling relationship between the strength of lipid metabolic gene pathway and CD8^+^ T cell infiltration/function, and survival outcome, which can be used to guide the priority of “metabolism-immune” combination (Simeth et al., 2025); radiopathomics, represented by the coupling of ADFP/PLIN2-CT HU, provides non-invasive lipid droplet/lipid metabolism readings (Deng et al., 2025); the combination of fluid metabolism fingerprints (plasma/urine) and AI models provides deployable companion diagnostics for early screening, efficacy prediction and follow-up. Tools (Ho et al., 2015; Ossolińska et al., 2025).

## 5 Continuous challenges and future prospects: prospects of precision metabolic oncology

### 5.1 Tumor–metabolic pathway plasticity of host and organ specificity

The plasticity of tumor metabolism makes it diverse in different microenvironments. Especially in ccRCC, the metabolic interactions between tumor cells and host organs (such as the liver and adipose tissue) remain poorly understood. Research shows that the metabolic characteristics of ccRCC are also closely linked to the host’s systemic metabolic status (such as obesity-related lipid mobilisation and diabetes-related hyperglycaemia) (Greco et al., 2024; Venkatesh et al., 2023). For example, the free fatty acids released by adipose tissue in obese patients may be taken up by ccRCC cells and used to synthesize lipid droplets or as an energy source, potentially affecting tumor aggressiveness and modifying therapeutic response (Yun et al., 2025; Niu et al., 2025b). Therefore, it is crucial to develop effective, individualised treatment strategies to understand metabolic interactions within the “tumor-host” ecosystem beyond the tumor itself.

### 5.2 Toxicity management and activation of metabolic adaptive bypass pathways

Metabolic reprogramming enables tumor cells to survive in harsh environments and may also improve treatment tolerance. For example, tumor cells may avoid the effect of drugs by changing their metabolic pathways, such as surviving by upregulating alternative amino acid metabolic pathways after using HIF-2α inhibitors, or resisting the oxidative stress caused by treatment by enhancing specific metabolic pathways (such as the synthesis of antioxidant glutathione) (Peng et al., 2025; Gavi et al., 2025; Li et al., 2024). This metabolic “detour” or redundancy is an essential mechanism for the development of acquired drug resistance, which also explains why single-targeted metabolic therapy is often effective (Li et al., 2024; Nho et al., 2025). The metabolic adaptive mechanism has introduced new challenges to treatment, and there is an urgent need to develop a joint treatment strategy that targets multiple key metabolic nodes simultaneously.

### 5.3 Insufficient evidence in the real world and the urgent need for cross-centre standardisation

There are still two core obstacles to translating findings on metabolic reprogramming into clinical practice. One is the lack of evidence level: at present, the scale and positive results of randomised controlled studies on metabolic targets are limited (for example, telaglenastat combined treatment fails to improve the outcome), and the mainstream guidelines also place it in the exploration stage (Tannir et al., 2022; Powles et al., 2024). The second is cross-centre repeatability and data governance: the metabolic group/lipid group needs to follow the sample processing and minimum report set of MSI and best practises, and the imaging end needs to unify the collection, reconstruction and feature definition according to the consensus of IBSI and machine learning to reduce batch differences and improve comparability (Sumner et al., 2007; Köfeler et al., 2021; Unterrainer et al., 2022) At the level of data co-construction, the FAIR principle should be implemented and federal learning should be adopted to achieve multi-institutional modelling under the premise of “not leaving the hospital” (Wilkinson et al., 2016; Sheller et al., 2020); at the same time, the study of tumor markers and diagnostic accuracy should follow REMARK/STARD and other reporting norms to improve external verifiability and Clinical promotion value (Sauerbrei et al., 2018; Bossuyt et al., 2015). Within the above-mentioned standardisation and governance framework, rigorously designed prospective, multi-centre clinical trials can confirm the real-world clinical value of metabolic targeting strategies.

### 5.4 Accurate decision-making tree driven by metabolic phenotype: DCCD/fat droplet spectrum, ferroptosis sensitivity, and HIF activity

Future research should focus on developing precise treatment strategies tailored to metabolic characteristics. For example, by analysing the metabolic phenotypes of tumor cells, such as DCCD status, lipid droplet accumulation, ferroptosis sensitivity, and HIF activity, an individualised treatment decision tree can be constructed (Hu et al., 2024; Dong et al., 2025). The preliminary decision-making framework could prioritize testing the “induced ferroptosis + ICI” scheme for “fat droplet enrichment/ferroptosis susceptibility” patients. In contrast, for “DCCD/fat droplet deficiency” patients, a combination of “HIF-2α inhibitor + targeted amino acid metabolism” may be required (Deng et al., 2025; Yang et al., 2025; He et al., 2024). These metabolic characteristics can serve as dynamic biomarkers to predict treatment response and drug resistance, providing a basis for truly individualised treatment.

### 5.5 Multimodal integration of AI, multi-omics, and metabolic imaging, and its clinical implementation

**FIGURE 1:**
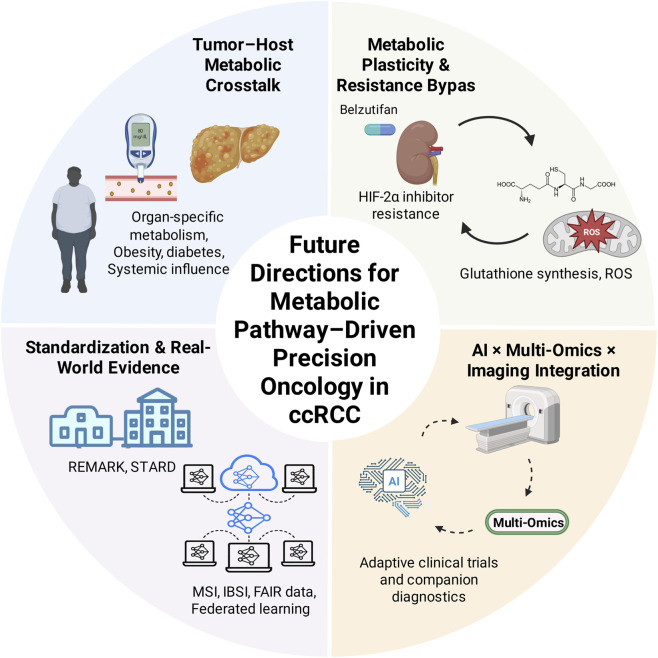
Future Directions in Precision Metabolic Oncology of ccRCC: Key points of the transformation of precision metabolic oncology in ccRCC. 1 Tumor-host metabolic interaction and plasticity; 2 Metabolic “detour” and drug resistance monitoring; 3 Standardisation and data governance (MSI/IBSI, FAIR, REMARK/STARD); 4 AI × multi-omics × imaging in stratification, prediction and experimental design.

The integration of artificial intelligence (AI) and multi-group data provides new perspectives and tools for the metabolic research of ccRCC. By combining genomics, transcriptomics, metabolomics, and imaging data, AI can help identify new metabolic markers and therapeutic targets (Dai et al., 2025; Zheng et al., 2025a; Chen et al., 2023). More importantly, AI can integrate heterogeneous data to build a comprehensive model that predicts a patient’s prognosis and treatment response (Chen et al., 2023; Zheng et al., 2025b). The progress of imaging metabolism enables the non-invasive, real-time monitoring of tumor metabolism, providing dynamic support for clinical decision-making. In the future, the integration of AI-driven multi-omics and imaging metabolism will be explicitly reflected in the development of “adaptive clinical trial design” and “intelligent companion diagnostics system”, thereby systematically promoting the clinical translation of ccRCC metabolism research. As shown in Figure 1, the plasticity of the tumor-host interaction and the metabolic reprogramming of metabolic pathways provide profound insights into the progression of ccRCC. In particular, in tumor-host metabolic interactions, host factors such as obesity and diabetes significantly affect tumor invasiveness and response to treatment. The metabolic adaptive bypass reveals how tumor cells escape drug treatment through metabolic reprogramming, providing new ideas for the development of precise treatment strategies.

## 6 Key priorities for clinical transformation

### 6.1 Precise patient stratification based on metabolic classification

Combined imaging–multi-omics markers can identify metabolic subtypes, such as “high glycolysis/low lipid-droplet load (including DCCD)/glutamine dependence,” which inform cohort stratification and efficacy prediction. The index is the objective remission rate of post-stratified first-line or combined treatment/progressive survival, which is significantly improved, and its repeatability has been reported (Fresnedo et al., 2025; Deng et al., 2025; Greco et al., 2024).

### 6.2 Accompanying the coordinated promotion of diagnosis and adaptive clinical trials

Starting in phase II, a threshold-locking companion diagnostic strategy was developed in parallel, and a basket/umbrella design with a medium-term suspension rule was implemented to enable rapid validation of the metabolic target. The indicators were the preset endpoint achievement rate, medium-term decision-making accuracy, and cross-centre consistency (Ho et al., 2015; Greco et al., 2024; Knott et al., 2018).

### 6.3 Data standardisation, model repeatability and cross-centre consistency

Collection, quality control, and sharing are carried out in accordance with MSI/IBSI and FAIR specifications. The indicators are the same effect direction of the cross-centre reproduction experiment, the performance difference within ≤ preset range, and the publicly available analysis process is formed (Greco et al., 2024; Choueiri et al., 2021; Zwanenburg et al., 2020).

## 7 Conclusion

ccRCC takes VHL/HIF inactivation as the molecular basis, forming a metabolic network characterised by enhanced sugar metabolism, lipid droplet/lipid remodelling, reprogramming of amino acid and one-carbon metabolism, and redox imbalance, and mutual traction with the immunosuppressive microenvironment, becoming a key driver of disease progression and treatment tolerance. This review integrates single-cell and spatial multiomics, metabolic/lipidomics, and imaging evidence, outlines the metabolic heterogeneity map of ccRCC and evolutionary pathways such as DCCD, and emphasises the close coupling of metabolic state and clinical outcomes. At the treatment level, strategies such as hypoxic axis inhibition, lipid suppression/induced ferroptosis, and glutamine and one-carbon metabolism provide a solid mechanistic basis and a transformational path for rational use with ICI/TKI/antivascular treatments. For clinical implementation, it is urgent to adopt the strategy of “metabolic phenotype-companion diagnostics-combined treatment” as the main line: use DCCD, lipid droplet/PLIN2 phenotype, ferroptosis sensitivity, and HIF activity as the stratification anchor points, and combine radiopathomics and body fluid metabolism fingerprints to complete the movement. State monitoring and efficacy prediction, and verification of replicable benefits in prospective, standardised, and multi-centre trials. Therefore, precise metabolic oncology is expected to become an essential pillar of individualised treatment for ccRCC.

## References

[B1] Almanzar G. Alarcon J. C. Garzon R. Navarro A. M. Ondo-Méndez A. Prelog M. (2025). Hypoxia and activation of hypoxia inducible factor alpha as influencers of inflammatory helper T cells in autoimmune disease - a link between cancer and autoimmunity. Front. Immunol. 2, 1633845. 10.3389/fimmu.2025.1633845 40963621 PMC12436505

[B2] Badoiu S. C. Greabu M. Miricescu D. Stanescu-Spinu I. I. Ilinca R. Balan D. G. (2023). PI3K/AKT/mTOR dysregulation and reprogramming metabolic pathways in renal cancer: crosstalk with the VHL/HIF axis. Int. J. Mol. Sci. 24, 8391. 10.3390/ijms24098391 37176098 PMC10179314

[B4] Bao M. Shi R. Zhang K. Zhao Y. Wang Y. Bao X. (2019). Development of a membrane lipid metabolism-based signature to predict overall survival for personalized medicine in ccRCC patients. EPMA J. 10 (4), 383–393. 10.1007/s13167-019-00189-8 31832113 PMC6882998

[B5] Bossuyt P. M. Reitsma J. B. Bruns D. E. Gatsonis C. A. Glasziou P. P. Irwig L. (2015). STARD 2015: an updated list of essential items for reporting diagnostic accuracy studies. Clin. Chem. 61, 1446–1452. 10.1373/clinchem.2015.246280 26510957

[B6] Brand A. Singer K. Koehl G. E. Kolitzus M. Schoenhammer G. Thiel A. (2016). LDHA-associated lactic acid production blunts tumor immunosurveillance by T and NK cells. Cell Metab. 24, 657–671. 10.1016/j.cmet.2016.08.011 27641098

[B8] Cancer Genome Atlas Research Network (2013). Comprehensive molecular characterization of clear cell renal cell carcinoma. Nature 499 (7456), 43–49. 10.1038/nature12222 23792563 PMC3771322

[B9] Chang C. H. Qiu J. O'Sullivan D. Buck M. D. Noguchi T. Curtis J. D. (2015). Metabolic competition in the tumor microenvironment is a driver of cancer progression. Cell 162, 1229–1241. 10.1016/j.cell.2015.08.016 26321679 PMC4864363

[B10] Chen S. Song D. Chen L. Guo T. Jiang B. Liu A. (2023). Artificial intelligence-based non-invasive tumor segmentation, grade stratification and prognosis prediction for clear-cell renal-cell carcinoma. Precis. Clin. Med. 6, pbad019. 10.1093/pcmedi/pbad019 38025974 PMC10680020

[B11] Choueiri T. K. Bauer T. M. Papadopoulos K. P. Plimack E. R. Merchan J. R. McDermott D. F. (2021). Inhibition of hypoxia-inducible factor-2α in renal cell carcinoma with belzutifan: a phase 1 trial and biomarker analysis. Nat. Med. 27, 802–805. 10.1038/s41591-021-01324-7 33888901 PMC9128828

[B12] Choueiri T. K. Powles T. Peltola K. de Velasco G. Burotto M. Suarez C. (2024). Belzutifan *versus* everolimus for advanced renal-cell carcinoma. N. Engl. J. Med. 391, 710–721. 10.1056/NEJMoa2313906 39167807

[B13] Dai H. Zhao K. Zhao Y. Jiang K. Hang Z. Huang X. (2025). Machine learning model in multi-omics perspective demystifies the prognostic significance of crotonylation heterogeneity in clear cell renal cell carcinoma. BMC Urol. 25, 229. 10.1186/s12894-025-01914-4 40954501 PMC12434922

[B14] Deng Q. Ji Y. Liu J. Wen T. (2025). Lipid reprogramming and ferroptosis crosstalk in clear cell renal cell carcinoma: metabolic vulnerabilities and therapeutic targeting. Mol. Cancer 24, 236. 10.1186/s12943-025-02457-w 41039547 PMC12492529

[B15] Dong M. Wang L. Hu N. Rao Y. Wang Z. Zhang Y. (2025). Integration of multi-omics approaches in exploring intra-tumoral heterogeneity. Cancer Cell Int. 25, 317. 10.1186/s12935-025-03944-2 40883767 PMC12395700

[B16] Fadini G. P. (2020). SGLT-2 inhibitors and circulating progenitor cells in diabetes. Cell Metab. 31, 883. 10.1016/j.cmet.2020.04.002 32302526

[B17] Fresnedo O. Lopez-Gomez J. A. Ceniceros C. Larrinaga G. Saiz A. Mosteiro L. (2025). Adaptations of lipid metabolism in low-grade clear cell renal cell carcinoma are linked to cholesteryl ester accumulation. Sci. Rep. 15, 24762. 10.1038/s41598-025-09664-x 40634478 PMC12241625

[B18] Gavi F. Sighinolfi M. C. Pallotta G. Assumma S. Panio E. Fettucciari D. (2025). Multiomics in renal cell carcinoma: current landscape and future directions for precision medicine. Curr. Urol. Rep. 26 (1), 44. 10.1007/s11934-025-01276-2 40418294 PMC12106551

[B19] Greco F. Panunzio A. Cerroni L. Cea L. Bernetti C. Tafuri A. (2024). CT characterization of lipid metabolism in clear cell renal cell carcinoma: relationship between liver hounsfield unit values and adipose differentiation-related protein gene expression. Int. J. Mol. Sci. 25, 12587. 10.3390/ijms252312587 39684299 PMC11640828

[B20] He C. Li Q. Wu W. Liu K. Li X. Zheng H. (2024). Ferroptosis-associated genes and compounds in renal cell carcinoma. Front. Immunol. 15, 1473203. 10.3389/fimmu.2024.1473203 39399506 PMC11466770

[B21] Ho P. C. Bihuniak J. D. Macintyre A. N. Staron M. Liu X. Amezquita R. (2015). Phosphoenolpyruvate is a metabolic checkpoint of anti-tumor T cell responses. Cell 162, 1217–1228. 10.1016/j.cell.2015.08.012 26321681 PMC4567953

[B22] Hu J. Wang S. G. Hou Y. Chen Z. Liu L. Li R. (2024). Multi-omic profiling of clear cell renal cell carcinoma identifies metabolic reprogramming associated with disease progression. Nat. Genet. 56, 442–457. 10.1038/s41588-024-01662-5 38361033 PMC10937392

[B23] Huang C. Wang G. Yuan Y. Zou Y. Tang X. Guo H. (2025). Development and validation of a novel plasma metabolomic signature for the detection of RCC. Eur. Urol. 10.1016/j.eururo.2025.09.4148 41047317

[B24] Jaakkola P. Mole D. R. Tian Y. M. Wilson M. I. Gielbert J. Gaskell S. J. (2001). Targeting of HIF-alpha to the von Hippel-Lindau ubiquitylation complex by O2-regulated prolyl hydroxylation. Science 292, 468–472. 10.1126/science.1059796 11292861

[B25] Jin J. Byun J. K. Choi Y. K. Park K. G. (2023). Targeting glutamine metabolism as a therapeutic strategy for cancer. Exp. Mol. Med. 55, 706–715. 10.1038/s12276-023-00971-9 37009798 PMC10167356

[B26] Jonasch E. Donskov F. Iliopoulos O. Rathmell W. K. Narayan V. K. Maughan B. L. (2021). Belzutifan for Renal Cell Carcinoma in von Hippel-Lindau Disease. N. Engl. J. Med. 385, 2036–2046. 10.1056/NEJMoa2103425 34818478 PMC9275515

[B27] Kaushik A. K. Tarangelo A. Boroughs L. K. Ragavan M. Zhang Y. Wu C. Y. (2022). *In vivo* characterization of glutamine metabolism identifies therapeutic targets in clear cell renal cell carcinoma. Sci. Adv. 8, eabp8293. 10.1126/sciadv.abp8293 36525494 PMC9757752

[B28] Klasson T. D. LaGory E. L. Zhao H. Huynh S. K. Papandreou I. Moon E. J. (2022). ACSL3 regulates lipid droplet biogenesis and ferroptosis sensitivity in clear cell renal cell carcinoma. Cancer Metab. 10, 14. 10.1186/s40170-022-00290-z 36192773 PMC9528056

[B29] Knott M. E. Manzi M. Zabalegui N. Salazar M. O. Puricelli L. I. Monge M. E. (2018). Metabolic footprinting of a clear cell renal cell carcinoma *in vitro* model for human kidney cancer detection. J. Proteome. Res. 17, 3877–3888. 10.1021/acs.jproteome.8b00538 30260228

[B30] Köfeler H. C. Ahrends R. Baker E. S. Ekroos K. Han X. Hoffmann N. (2021). Recommendations for good practice in MS-based lipidomics. J. Lipid. Res. 62, 100138. 10.1016/j.jlr.2021.100138 34662536 PMC8585648

[B31] Li Y. Sun X. X. Qian D. Z. Dai M. S. (2020). Molecular crosstalk between MYC and HIF in cancer. Front. Cell Dev. Biol. 8, 590576. 10.3389/fcell.2020.590576 33251216 PMC7676913

[B32] Li K. Zhu Y. Cheng J. Li A. Liu Y. Yang X. (2023). A novel lipid metabolism gene signature for clear cell renal cell carcinoma using integrated bioinformatics analysis. Front. Cell Dev. Biol. 11, 1078759. 10.3389/fcell.2023.1078759 36866272 PMC9971983

[B33] Li M. Wang Y. Wei X. Cai W. F. Wu J. Zhu M. (2024). AMPK targets PDZD8 to trigger carbon source shift from glucose to glutamine. Cell Res. 34, 683–706. 10.1038/s41422-024-00985-6 38898113 PMC11442470

[B34] Lin R. Zhang H. Yuan Y. He Q. Zhou J. Li S. (2020). Fatty acid oxidation controls CD8+ tissue-resident memory T-cell survival in gastric adenocarcinoma. Cancer Immunol. Res. 8, 479–492. 10.1158/2326-6066.CIR-19-0702 32075801

[B35] Lyu H. Bao S. Cai L. Wang M. Liu Y. Sun Y. (2025). The role and research progress of serine metabolism in tumor cells. Front. Oncol. 15, 1509662. 10.3389/fonc.2025.1509662 40265021 PMC12011608

[B36] Nezami B. G. MacLennan G. T. (2024). Clear cell renal cell carcinoma: a comprehensive review of its histopathology, genetics, and differential diagnosis. Int. J. Surg. Pathol. 33, 265–280. 10.1177/10668969241256111 39051572

[B37] Nho S. B. Do S. H. Oh S. Park Y. C. Kim S. K. (2025). Enhanced glutathione production by a non-GMO Saccharomyces cerevisiae mutant isolated *via* acrolein resistance-mediated screening. Food Sci. Biotechnol. 34 (16), 3969–3978. 10.1007/s10068-025-01995-9 41211560 PMC12589744

[B38] Niu C. Wei H. Pan X. Wang Y. Song H. Li C. (2025a). Foxp3 confers long-term efficacy of chimeric antigen receptor-T cells *via* metabolic reprogramming. Cell Metab. 37, 1426–1441.e7. 10.1016/j.cmet.2025.04.008 40328248

[B39] Niu Q. Mou Y. Yao Y. Dong H. Wang K. Zeng Z. (2025b). Multidimensional analysis reveals the potential of ACSL3 as a cancer biomarker: from pan-cancer exploration to functional validation in hepatocellular carcinoma. Clin. Exp. Med. 25, 351. 10.1007/s10238-025-01882-x 41171313 PMC12578706

[B40] Ossolińska A. Płaza-Altamer A. Ossoliński K. Ossoliński T. Ruman T. Nizioł J. (2025). Untargeted metabolomic profiling of serum and urine in kidney cancer: a non-invasive approach for biomarker discovery. Metabolomics 21, 97. 10.1007/s11306-025-02294-4 40593405 PMC12213972

[B41] Peng C. Zhang F. Zhou F. Tan S. Xie W. (2025). LncRNA HCP5: a key regulator of tumor metabolic reprogramming, signaling pathway modulation, and therapeutic resistance. Int. J. Biol. Macromol. 27, 148606. 10.1016/j.ijbiomac.2025.148606 41161440

[B42] Powles T. Albiges L. Bex A. Comperat E. Grünwald V. Kanesvaran R. (2024). Renal cell carcinoma: ESMO clinical practice guideline for diagnosis, treatment and follow-up. Ann. Oncol. 35, 692–706. 10.1016/j.annonc.2024.05.537 38788900

[B43] Powles T. Choueiri T. K. Albiges L. Peltola K. de Velasco G. Burotto M. (2025). Health-related quality of life with belzutifan *versus* everolimus for advanced renal cell carcinoma (LITESPARK-005): patient-reported outcomes from a randomised, open-label, phase 3 trial. Lancet. Oncol. 26, 491–502. 10.1016/S1470-2045(25)00032-4 40112850

[B44] Reinfeld B. I. Rathmell W. K. Kim T. K. Rathmell J. C. (2022). The therapeutic implications of immunosuppressive tumor aerobic glycolysis. Cell Mol. Immunol. 19, 46–58. 10.1038/s41423-021-00727-3 34239083 PMC8752729

[B45] Ren X. Wang X. Zheng G. Wang S. Wang Q. Yuan M. (2024). Targeting one-carbon metabolism for cancer immunotherapy. Clin. Transl. Med. 14, e1521. 10.1002/ctm2.1521 38279895 PMC10819114

[B46] Sauerbrei W. Taube S. E. McShane L. M. Cavenagh M. M. Altman D. G. (2018). Reporting recommendations for tumor marker prognostic studies (REMARK): an abridged explanation and elaboration. J. Natl. Cancer Inst. 110, 803–811. 10.1093/jnci/djy088 29873743 PMC6093349

[B47] Sheller M. J. Edwards B. Reina G. A. Martin J. Pati S. Kotrotsou A. (2020). Federated learning in medicine: facilitating multi-institutional collaborations without sharing patient data. Sci. Rep. 10, 12598. 10.1038/s41598-020-69250-1 32724046 PMC7387485

[B48] Shi R. Sun J. Zhou Z. Shi M. Wang X. Gao Z. (2025). Integration of multiple machine learning approaches develops a gene mutation-based classifier for accurate immunotherapy outcomes. NPJ Precis. Oncol. 9 (1), 54. 10.1038/s41698-025-00842-8 40011681 PMC11865301

[B49] Simeth J. Engelmann S. Mayr R. Kaelble S. Weber F. Pichler R. (2025). Lipid metabolism of clear cell renal cell carcinoma predicts survival and affects intratumoral CD8 T cells. Transl. Oncol. 61, 102513. 10.1016/j.tranon.2025.102513 40902455 PMC12444183

[B51] Song G. Xue S. Zhu Y. Wu C. Ji X. (2024). The efficacy and safety of belzutifan inhibitor in patients with advanced or metastatic clear cell renal cell carcinoma: a meta-analysis. BMC Pharmacol. Toxicol. 25, 100. 10.1186/s40360-024-00828-5 39707485 PMC11660830

[B52] Sumner L. W. Amberg A. Barrett D. Beale M. H. Beger R. Daykin C. A. (2007). Proposed minimum reporting standards for chemical analysis Chemical analysis working group (CAWG) metabolomics standards initiative (MSI). Metabolomics 3, 211–221. 10.1007/s11306-007-0082-2 24039616 PMC3772505

[B53] Sung H. Ferlay J. Siegel R. L. Laversanne M. Soerjomataram I. Jemal A. (2021). Global cancer statistics 2020: GLOBOCAN estimates of incidence and mortality worldwide for 36 cancers in 185 countries. CA Cancer J. Clin. 71, 209–249. 10.3322/caac.21660 33538338

[B54] Tannir N. M. Agarwal N. Porta C. Lawrence N. J. Motzer R. McGregor B. (2022). Efficacy and safety of telaglenastat plus cabozantinib vs placebo plus cabozantinib in patients with advanced renal cell carcinoma: the CANTATA randomized clinical trial. JAMA Oncol. 8, 1411–1418. 10.1001/jamaoncol.2022.3511 36048457 PMC9437824

[B55] Unterrainer M. Deroose C. M. Herrmann K. Moehler M. Blomqvist L. Cannella R. (2022). Imaging standardisation in metastatic colorectal cancer: a joint EORTC-ESOI-ESGAR expert consensus recommendation. Eur. J. Cancer 176, 193–206. 10.1016/j.ejca.2022.09.012 36274570

[B56] Venkatesh N. Martini A. McQuade J. L. Msaouel P. Hahn A. W. (2023). Obesity and renal cell carcinoma: biological mechanisms and perspectives. Semin. Cancer Biol. 94, 21–33. 10.1016/j.semcancer.2023.06.001 37286114 PMC10526958

[B57] Wang M. (2023). Targeting glutamine use in RCC. Nat. Rev. Nephrol. 19, 151. 10.1038/s41581-023-00684-2 36694057

[B58] Wang W. Zhang X. Jiang S. Xu P. Chen K. Li K. (2023). A novel signature constructed by differential genes of muscle-invasive and non-muscle-invasive bladder cancer for the prediction of prognosis in bladder cancer. Front. Immunol. 14, 1187286. 10.3389/fimmu.2023.1187286 37691944 PMC10483405

[B59] Wang W. Shen J. Song D. Fu K. Fu X. (2024). Identification of macrophage-related genes in bladder cancer patients using single-cell sequencing and construction of a prognostic model. Am. J. Clin. Exp. Immunol. 13, 88–104. 10.62347/VLDZ7581 39022795 PMC11249859

[B60] Wang H. Xiao T. Zhuang H. Liu Y. Jin K. Li J. (2025). STBD1 mediates the crosstalk between glycogen and lipid droplets in clear cell renal cell carcinoma. Cell Rep. 44, 116429. 10.1016/j.celrep.2025.116429 41105508

[B61] Watson M. J. Vignali P. D. A. Mullett S. J. Overacre-Delgoffe A. E. Peralta R. M. Grebinoski S. (2021). Metabolic support of tumor-infiltrating regulatory T cells by lactic acid. Nature 591, 645–651. 10.1038/s41586-020-03045-2 33589820 PMC7990682

[B75] Wild C. P. Weiderpass E. Stewart B. W. (Editors). (2020). World Cancer Report: Cancer research for cancer prevention. Lyon, FR: International Agency for Research on Cancer. 39432694

[B62] Wilkinson M. D. Dumontier M. Aalbersberg I. J. Appleton G. Axton M. Baak A. (2016). The FAIR guiding principles for scientific data management and stewardship. Sci. Data. 3, 160018. 10.1038/sdata.2016.18 26978244 PMC4792175

[B63] Yang L. Wang X. Liu J. Liu X. Li S. Zheng F. (2023). Prognostic and tumor microenvironmental feature of clear cell renal cell carcinoma revealed by m6A and lactylation modification-related genes. Front. Immunol. 14, 1225023. 10.3389/fimmu.2023.1225023 37638005 PMC10450969

[B64] Yang G. Cheng J. Xu J. Shen C. Lu X. He C. (2024). Metabolic heterogeneity in clear cell renal cell carcinoma revealed by single-cell RNA sequencing and spatial transcriptomics. J. Transl. Med. 22, 210. 10.1186/s12967-024-04848-x 38414015 PMC10900752

[B65] Yang J. Miao D. Li X. Zhao C. Tan D. Wu S. (2025). Emerging roles of metabolic biomarkers in renal cell carcinoma: from molecular mechanisms to clinical implications. Front. Cell Dev. Biol. 13, 1664292. 10.3389/fcell.2025.1664292 41063965 PMC12500641

[B66] Yecies J. L. Manning B. D. (2011). Transcriptional control of cellular metabolism by mTOR signaling. Cancer Res. 71, 2815–2820. 10.1158/0008-5472.CAN-10-4158 21487041 PMC3693464

[B67] Yun J. E. Seo J. Koh J. Im S. A. Hong K. Y. Son Y. (2025). Cancer manipulates adjacent adipose tissue to exploit fatty acids *via* HIF-1α/CCL2/PPARα axis: a metabolic circuit to support tumor progression. Adv. Sci. (Weinh)., e15186. 10.1002/advs.202515186 41160815 PMC12806468

[B68] Zhu H. Wang X. Lu S. Ou K. (2023). Metabolic reprogramming of clear cell renal cell carcinoma. Front. Endocrinol. 14, 1195500. 10.3389/fendo.2023.1195500 37347113 PMC10280292

[B69] Zhang Y. Zhang S. Sun H. Xu L. (2025a). The pathogenesis and therapeutic implications of metabolic reprogramming in renal cell carcinoma. Cell Death Discov. 11, 186. 10.1038/s41420-025-02479-9 40253354 PMC12009291

[B70] Zhang H. Fan J. Kong D. Sun Y. Zhang Q. Xiang R. (2025b). Immunometabolism: crosstalk with tumor metabolism and implications for cancer immunotherapy. Mol. Cancer 24, 249. 10.1186/s12943-025-02460-1 41063213 PMC12505609

[B71] Zheng Q. Mei H. Weng X. Yang R. Jiao P. Ni X. (2025a). Artificial intelligence-based multimodal prediction for nuclear grading status and prognosis of clear cell renal cell carcinoma: a multicenter cohort study. Int. J. Surg. 111, 3722–3730. 10.1097/JS9.0000000000002368 40146270 PMC12165539

[B72] Zheng Q. Wei L. Zhou Y. Yang R. Jiao P. Mei H. (2025b). An artificial intelligence model for nuclear grading of clear cell renal cell carcinoma using whole slide images: a retrospective, multicenter, diagnostic study. Int. J. Surg. 111, 4400–4411. 10.1097/JS9.0000000000002484 40358632

[B73] Zhou Q. Meng Y. Li D. Yao L. Le J. Liu Y. (2024). Ferroptosis in cancer: from molecular mechanisms to therapeutic strategies. Signal Transduct. Target. Ther. 9, 55. 10.1038/s41392-024-01769-5 38453898 PMC10920854

[B74] Zwanenburg A. Vallières M. Abdalah M. A. Aerts H. J. W. L. Andrearczyk V. Apte A. (2020). The image biomarker standardization initiative: standardized quantitative radiomics for high-throughput image-based phenotyping. Radiology 295, 328–338. 10.1148/radiol.2020191145 32154773 PMC7193906

